# The Intriguing Blindness of Maria Theresia von Paradis and the Instructive Potential of Medical Biography

**DOI:** 10.7759/cureus.78948

**Published:** 2025-02-13

**Authors:** Curtis E Margo, Lynn E Harman

**Affiliations:** 1 Departments of Ophthalmology, Pathology and Cell Biology, University of South Florida Health Morsani College of Medicine, Tampa, USA; 2 Department of Ophthalmology, University of South Florida Health Morsani College of Medicine, Tampa, USA

**Keywords:** autism disorder, feigned symptoms, franz mesmer, functional vision loss, hysterical blindness, maria paradis, maria theresia von paradis, medical history

## Abstract

Medical biography can introduce healthcare providers to clinically relevant subjects that are rarely encountered in traditional curricula. Maria Theresia von Paradis (1759-1824), the blind musical prodigy, faced doubt about her reputation because of a brief and questionable recovery of sight at the age of 18 years. Her story serves as an example of how extraordinary circumstances can be met with skepticism. Her miraculous return of vision 15 years after going blind was mediated and exploited in public demonstrations by Franz Mesmer, a promoter of the theory of animal magnetism. Maria’s sight was lost a second time in 1778 just before Mesmer was driven from medical practice as a charlatan. Although the cause of her visual disability will never be known, it deviates from patterns of functional (hysterical) vision loss by its early age of onset, severity, and length of affliction. Paradis’s early proficiency in playing musical instruments raises questions about savant syndrome, autism, feigned disability, and how the early loss of vision influences aspects of cognitive development.

## Introduction and background

Investigation of disorders affecting historical figures can shed light on medical conditions and medical practices seldom discussed in traditional curricula. We use the narrative of Maria Theresia von Paradis (1759-1824), an accomplished Viennese musician and composer, to illustrate how medical biography can expose healthcare providers to important and interrelated topics in medicine that are often overlooked during training. Maria Theresia von Paradis rose to fame early in life for having mastered the organ, harpsichord, and piano without the benefit of sight. Her reputation as a musical genius who overcame blindness has been questioned in modern times because of the improbability of having temporally regained vision as a teenager while under spurious medical care. This historical review serves as a pedagogical tool to prompt reflection on topics ranging from hysterical blindness and feigned vision loss to autism, musical savant, and how early onset blindness affects the cognitive development of the brain.

## Review

Sources

The sources for the historical narrative used to analyze this medical case were drawn from popular literature, motion pictures (e.g., Licht directed by Barbara Albert, Prime Video, 2017; Mesmer directed by Robert Spottiswoode, 1994), and multiple websites that describe the life and career of Maria Theresia von Paradis (e.g., Wikipedia, Museum of Music History, Classic FM Music, Encyclopedia.com) [1,2]. Additional sources included authors who have written about Maria Theresia von Paradis as an inspirational figure for the blind [3,4]. This retrospective clinical analysis was based on literature searches using the following terms: hysterical blindness, feigned vision loss, musical savant, and their common synonyms.

Historical narrative

*Maria Theresia von Paradis* *and Blindness* 

Maria Theresia von Paradis was born a healthy child on May 15, 1759. Before the age of four years, she was blind. Her father Joseph Anton was Imperial Secretary of Commerce and Court Councilor to Empress Maria Theresa (1717-1780), the autocratic ruler of the Habsburg dominion. It is unclear if naming his child after the sovereign may have ingratiated Joseph with the ruler. Years later when the monarch learned that a bureaucrat’s child with precocious faculty for music had gone unexpectedly blind, she granted the family a generous pension [5]. Since the onset of blindness, Maria Theresia von Paradis was examined by ocular specialists, who diagnosed paralysis of the optic nerves and had nothing to offer other than sympathy. Her amazing ability to play keyboard instruments without sight led to Paradis being praised and recognized as a genius [5].

Her life became more complicated at age 17 when her parents took her to Franz Anton Mesmer (1734-1815), a physician who developed therapies based on a theory of animal magnetism [6]. Once under the care of Mesmer, he treated her with magnets and magician-like hand and arm motions. The peculiar therapy accomplished a miracle in short order. Some measure of vision was restored to a teenager who had presumably not seen for 15 years. The prestige of achieving this therapeutic triumph was not lost on Mesmer, who set forth to exhibit his accomplishment to the public. He had Maria Theresia von Paradis perform visual tasks in front of small audiences, where she would identify various objects in the room [1]. To allay concerns that the performance was rigged, audience members were allowed to test her responses to hand signals or assess her credibility in other ways. Maria may have sensed that people did not believe she had ever been blind, which led her to become distraught. As a result, her piano recitals began to suffer [1].

**Figure 1:**
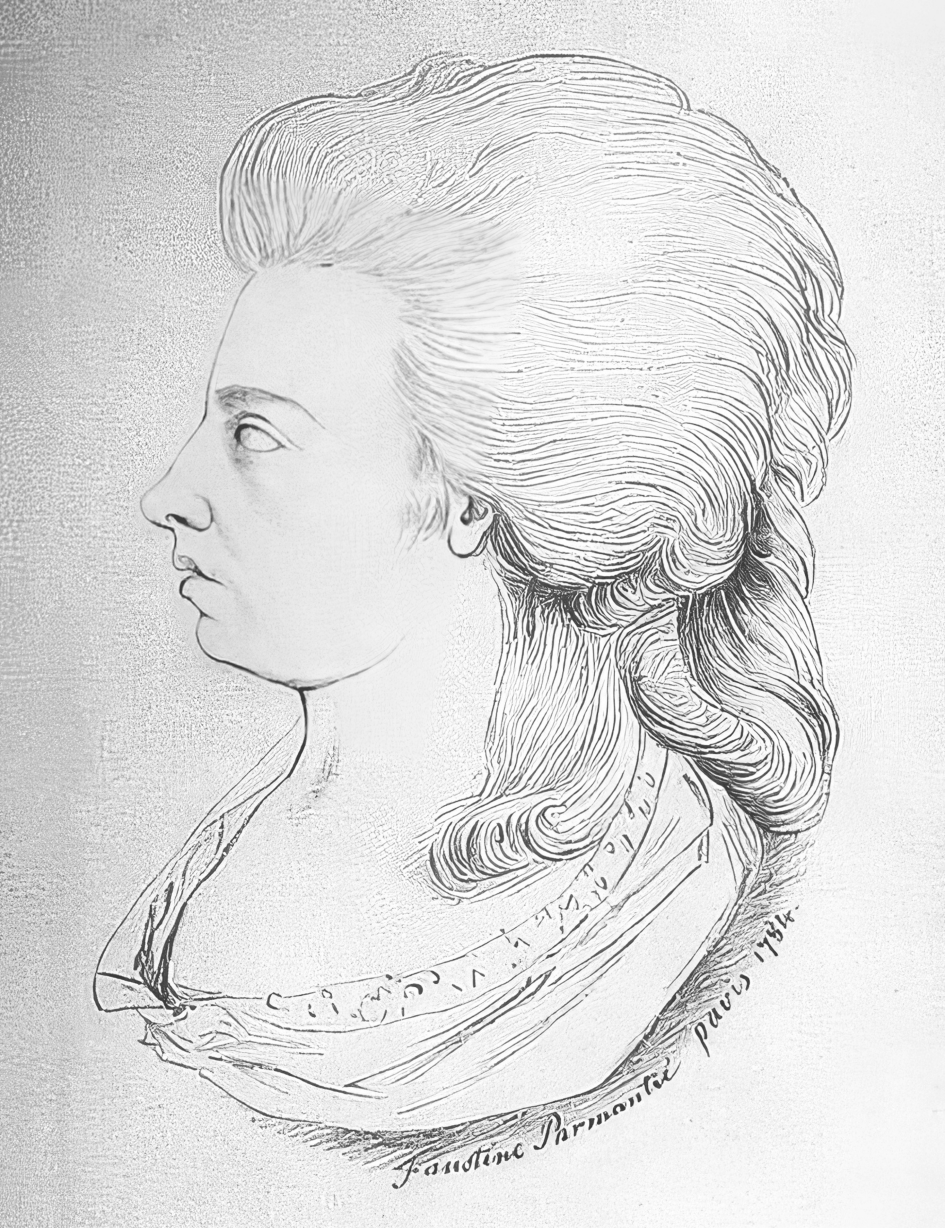
Portrait of Maria Theresia von Paradis. Portrait of Maria Theresia von Paradis at the age of 25 years by Faustine Parmantié. She had no documented ocular deformity or “wobbly” eyes (nystagmus). She was acknowledged for being a good dancer and having fine taste in clothing. Maria Theresia von Paradis portrait in Wikipedia. The work is in the public domain.

Her miraculous recovery of vision was short-lived. Stories portrayed in literature and in motion pictures depict her waning vision as brought on by fear her parents would lose their government pension, or by a brooding anxiety that she would be separated from Dr. Mesmer with whom she had become emotionally dependent [1]. Her interlude with vision was fleeting; she would remain blind until her death at the age of 65 years. Her musical competence, however, soon returned and she enjoyed a productive career as a performer and composer, touring much of Europe (Figure 1) [7].

Franz Anton Mesmer

**Figure 2:**
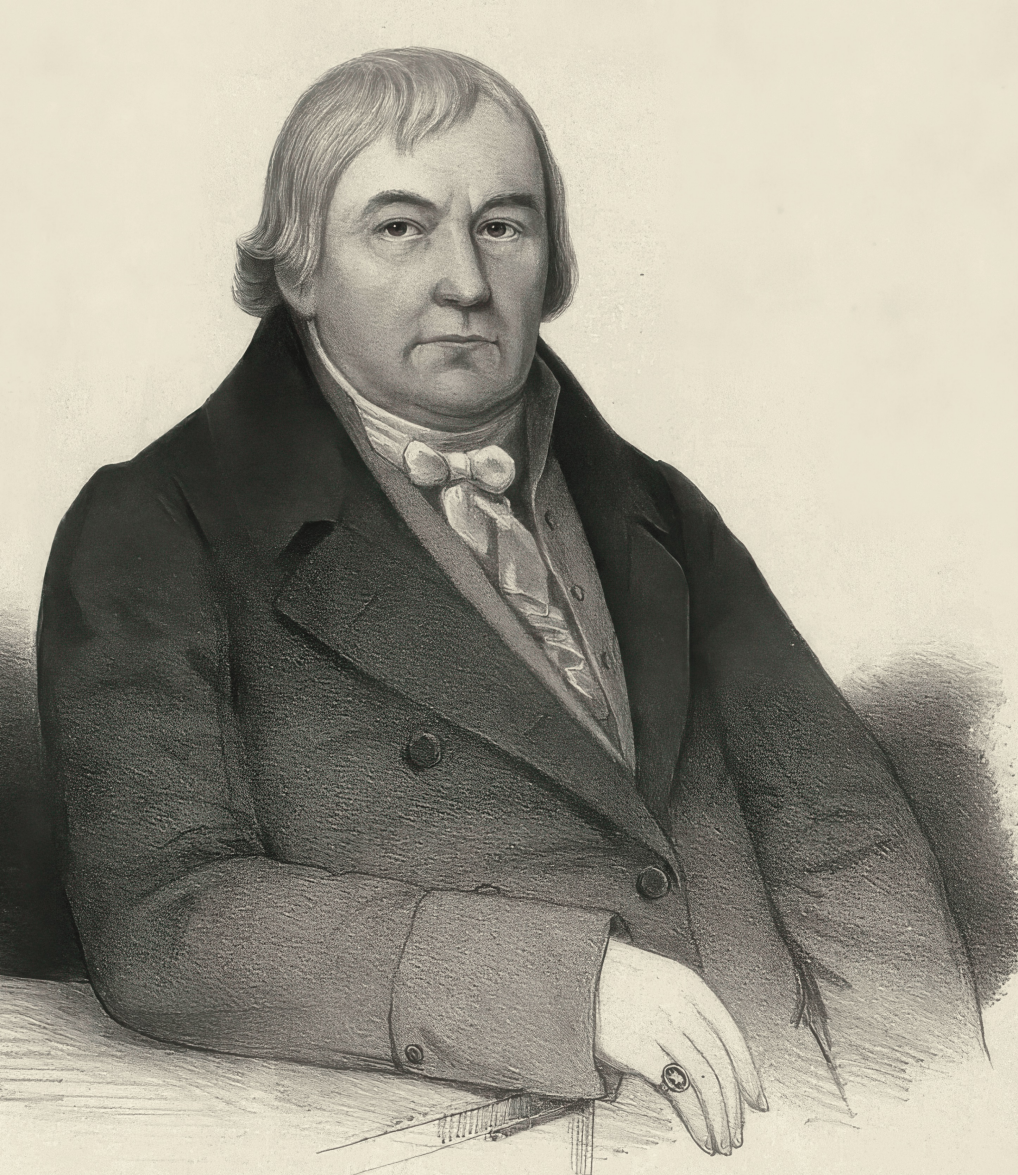
Portrait of Franz Mesmer. Lithograph of a portrait by an unknown artist of Franz Anton Mesmer painted at the age of 66 years. By this time he had been driven off the continent for his scandalous medical practices, but his technique of inducing trancelike states was the forerunner of hypnosis. Courtesy Österreichische Nationalbibliothek, Vienna. This work is in the public domain.

The events surrounding the treatment of Maria Theresia von Paradis reinforced local medical opinion that Mesmer was a con artist [1]. Franz Anton Mesmer (1734-1815) came from a family of modest means. His father was a gamekeeper in a small Swabian village. He was a curious and imaginative child. As a young man, he studied theology and law at the university before turning his creative instincts to medicine, receiving his medicine degree from the University of Vienna in 1766. During medical school, he completed a dissertation on how the planets influenced the human body in much the same way the gravitational forces of the Moon influenced the tides on Earth [6]. This conceptual equivalence was the foundation of his animal magnetism theory. The invisible forces of magnets fascinated Mesmer so much that he began to test their therapeutic powers on patients [6]. Two years after medical school, he married Anna Maria von Posch, a wealthy widow who owned a large estate on the banks of the Danube River. With few financial concerns, he devoted more time studying animal magnetism (Figure 2) [8]. Mesmer interpreted his results as confirmation of his passionately held opinion that magnetic forces influenced health and disease. As his experience treating patients grew, the role that magnets played became secondary to the manner in which he moved his hands and arms during each treatment session [1,6]. After Maria Theresia von Paradis relapsed, the medical community in Vienna grew increasingly concerned that Mesmer was a charlatan. In 1778, he was pressured to abandon his clinical practice and fled to Paris [6].

Historical analysis

Maria Theresia von Paradis’s life and sketchy ocular history raise fascinating questions about functional blindness, feigned vision loss, musical savant, autism, and how congenital or early onset blindness affects neuroplasticity [1,9,10]. 

Functional Blindness and Hysterical Amblyopia

The implausible return of Paradis’s vision after years of blindness did not appear to raise concerns over the nature of her eye disease or her state of mental health as it might today. Much of this can be attributed to the lack of knowledge at the time about the natural history of eye diseases. In a similar sense, issues of mental illness did not reach a threshold of concern until signs of overt madness, or horrific uncontrolled mood swings were manifest. Curiosity over what eye or mental disorder may have affected Maria Theresia von Paradis is a modern fascination [1-4,9,10].

The claim of functional or feigned vision loss is doubtful. Her father noted Maria “blind” on December 9, 1762, when she was just three years seven months of age. It lasted her lifetime, except for the brief respite at the age of 18 years. The concept of hysterical amblyopia, or psychological blindness caused by emotional stress (later called conversion reaction), was still decades away. By the early part of the 20th century, non-organic vision loss (not due to structural disease) was known to cause symptoms of central vision loss, night blindness, disturbances in visual fields, dyschromatopsia, double vision, or photophobia [11,12]. Feigned vision loss was appreciated later when faked blindness was associated with conspicuous secondary gain [13].

In children, the vast majority of functional vision loss is detected after the age of five years [11,12]. Visual symptoms are usually mild-to-moderate and show signs of improvement or resolve within weeks to months [11-18]. Rarely does functional vision loss decline enough to impair ambulation, and in only a few cases has the disability persisted beyond a few years. Maria Theresia von Paradis’ medical and vision history is woefully meager. However, from what is known of her vision loss in terms of early age of onset, severity, and longevity makes the diagnosis of functional or feigned vision loss untenable. There is no modern medically documented case describing such an experience.

*Early Onset Blindness* 

Assuming that the visual impairment of Paradis was bilateral, severe, and detected at three years of age, it limits diagnostic possibilities. Two potential retinal disorders are Leber’s congenital amaurosis (not to be confused with Leber’s hereditary optic neuropathy) and early-onset severe retinal dystrophy. Both conditions occur in girls and boys with equal frequency and can present at an early age with profound visual impairment [19]. The phenotypic expression has been widening and can present without systemic co-pathologies. Another consideration would be dominant optic atrophy, which unlike Leber’s hereditary optic atrophy affects girls and boys equally. Its phenotypic expression is also broad and can manifest as an isolated disorder of vision [20]. Other uncommon eye diseases of early childhood could fit her clinical profile, but speculating on etiology may not be as important as the possibility that Paradis may have had subnormal vision rather than totally extinguished perception. The lay use of the term “blind” lacks medical precision, particularly before the 19th century when the idea of measuring levels of vision was nascent [21].

Early Onset Visual Impairment

Though Maria Theresia von Paradis conducted herself in public settings as blind, there are several observations suggesting she had some vision. When she was first introduced to Mesmer at a social gathering, he noted she could “dance the minuet to perfection” [5]. The minuet is a couples dance, choreographed and embellished with bows and curtsies, activities nearly impossible to command with poise without some element of sight. Paradis was also fashion-conscious and a recognized stylish dresser, something unusual for an individual supposedly “blind” since the age of three years [5]. Fine visual acuity is not required to master choreographed dance or to appreciate the latest fashion designs, but without visual clues or visual experiences, these are odd preoccupations for the sightless. It is conceivable that Maria Paradis had seriously compromised vision (i.e., low vision) since the distinction between subnormal vision and “blindness” was immaterial in 18th-century Europe [21].

The Great Mesmerizer

After fleeing Vienna, Mesmer discovered his talents were in demand in Paris. His clinic waiting room was filled with an assortment of patients, almost all of whom were managed with the same theatrical manipulations he perfected in Austria to restore magnetic balance. Much of his success with clients was due to his demeanor and persuasive personality. He learned that his methodical words and gentle hand motions could induce a strange sleeplike state called somnambulism in a subset of patients [6]. This ability was appreciated by one of his apprentices, the Marquis de Puységur (1731-1825), who saw great potential in placing patients into a trance-like state. He actively pursued the art and further refined it. The technique, known as mesmerism, was propagated by local therapists. It was the forerunner of hypnosis and used by the “father” of neurology Jean-Martin Charcot (1825-1893) to study hysteria [6].

Mesmer may have sensed that Paradis was not totally blind after he first met her by noting her graceful dancing and good taste in clothes. It is possible that the worldly doctor plied his magnetic personality to coax a poor-sighted teenager into displaying her residual vision to further his professional career. Alternatively, Mesmer could have convinced a naive truly blind 18-year-old to participate in staged performances. If she was a co-conspirator in rigged demonstrations, it is conceivable that guilt played a role in her unanticipated emotional collapse soon after her vision was supposedly restored. 

Musical Genius

Could the absence of normal vision have anything to do with the exceptional prowess Paradis demonstrated on the keyboard? The early deprivation of normal visual sensory input is known to augment other neurodevelopmental pathways in a process referred to as cross-modal plasticity [22]. Congenital or early loss of sight leads to considerable remodeling of neural networks, potentially enhancing other cortical systems. In behavioral research, this type of neuroplasticity has also been called paradoxical functional facilitation [23]. It is paradoxical only in the sense that the neural damage (e.g., blindness) results in the promotion of other cognitive modalities. Studies have shown the neuroanatomical effects of early blindness are profound with estimates that over 25% of the human cortex is altered in various ways following the loss of vision [24,25]. It is well established that the occipital cortex in the congenitally blind is reconfigured to deal with a variety of different sensory stimuli and cognitive tasks [25]. The phenomenal ability to naturally remodel neuronal networks in young brains is seen by researchers not as disabling but rather as creating a “differently able brain” [25]. In terms of the performing arts, blindness can be associated with the heightening of various behavioral/cognitive competencies like music [26].

A Savant Connection

Was the musical talent of Maria Theresia von Paradis due to something beyond simple cross-modal plasticity? Could she have been a savant, in which her musical endowments far eclipsed that attributable to general aptitude and intelligence? [25,26]. Any discussion of extraordinary talent is complicated by the subjectivity involved in determining it. The concept of a savant is usually understandable in the context of domain exceptionalism coexisting with general cognitive impairment (e.g., calendar calculations in someone who cannot grasp algebra) [27,28]. These so-called “idiotic savants” have included individuals gifted in art, mathematical calculations, and music. In terms of musical exceptionalism, children are often self-taught at a young age - like Paradis. She, however, had a range of other interests and skills and displayed no evidence of global cognitive impairment. She learned other languages and taught herself to read using embossed letters, which in later life she encouraged caretakers of the blind to teach to their wards [10]. 

The Autism Question

With the marked increase in the prevalence of autism, few extremely gifted individuals escape suspicion as falling along the spectrum. Psychologists estimate as many as 37% of extraordinarily gifted persons have traits of autism, which may expand further as the autistic phenotype widens [28]. As currently perceived, there is a connection between visual impairment and autism [29]. Autism spectrum disorder is found 30 times more often in children with congenital blindness [30]. Such profoundly elevated risk typically indicates a causal connection, as does the observation that children with autism have structural and functional abnormalities of sensory brain systems, particularly the visual system [30].

Maria Theresia von Paradis, however, never demonstrated any of the defining features of autism. She enjoyed social interactions with friends and acquaintances. She had interests beyond music and actively participated in social issues, such as improving the plight of others suffering from blindness [1-6,9,10].

Summary 

The inexplicable return of vision at the age of 18 years may have potentially blemished the reputation of Maria Theresia von Paradis, whose musical proficiency as a visually impaired person made her a heroic female figure. It is unlikely that she suffered from hysterical amblyopia or feigned vision loss given no medically documented patient with comparable lifetime impairment has been reported in the literature. One plausible explanation is that she was blind or perhaps retained a bit of subnormal vision and Franz Mesmer convinced the teenage Paradis to pretend she could see normally. Her fascinating but unresolvable medical history raises awareness of seldom discussed subjects, including spurious medical therapies, medical con artists, feigned vision loss, hysterical conversion reaction, autism, and the medical designation of savant. 

## Conclusions

There is no historical precedent for hysterical blindness or feigned vision loss starting at the age of three years and lasting a lifetime. Maria Theresia von Paradis likely had subnormal vision or was totally blind and Messmer coaxed her into faking vision. This historical perspective solicits inquiry into related topics of musical savant and the relationship of blindness with autism. There is undoubtedly some neurobiological basis for musical “genesis” but what defines that mix of original creativity, auditory perception, memory, and physical dexterity is nearly impossible to define. Savant has the potential for misapplication unless used in the context of persons with a focused area of genius coexisting with impoverished intellectual ability. The diagnosis of autism needs to be applied cautiously in children who are blind since so many so-called autistic traits are behaviors usually learned through visual experiences. Maria Theresia von Paradis was a top-caliber musician, but she was neither a savant in a medical sense nor displayed evidence of autism. Although a medical diagnosis will never be known with certainty, her historical narrative serves as a pedagogical tool that invites discussion about a variety of interrelated topics in medicine, including how the medical profession should deal with con artists.
